# Multisystemic and cerebral infection associated with *Halicephalobus gingivalis* in Brazilian Criollo horses

**DOI:** 10.1007/s11259-026-11522-8

**Published:** 2026-09-29

**Authors:** Laura Berger, Joanna V. Z. Echenique, Vinícius Baggio-Souza, Renata Fagundes-Moreira, Paula R. Ribeiro, Francisca M. S. Barbosa, Eryca C. Lamego, Flávia U. Bueno, Saulo P. Pavarini, Luciana Sonne, Welden Panziera, João F. Soares

**Affiliations:** 1 Laboratório de Protozoologia e Rickettsioses Vetoriais – ProtozooVet, Faculdade de Veterinária, Universidade Federal do Rio Grande do Sul, Avenida Bento Gonçalves, Porto Alegre, Agronomia 9090 Brazil; 2 Minnesota Veterinary Diagnostic Laboratory, University of Minnesota, 1333 Gortner Ave, St Paul, MN 55108 USA; 3 Department of Veterinary Medicine, University of Bari, Valenzano, 70010 Italy; 4 PhD National Programme in One Health Approaches to Infectious diseases and Life Science Research, Department of Public Health, Experimental ad Forensice Medicine, University of Pavia, Pavia, 27100 Italy; 5 Setor de Patologia Veterinária, Faculdade de Veterinária, Universidade Federal do Rio Grande do Sul, Avenida Bento Gonçalves, Porto Alegre, Agronomia 9090 Brazil; 6 Faculdade de Veterinária, Hospital de Clínicas Veterinárias, Universidade Federal do Rio Grande do Sul, Avenida Bento Gonçalves, Porto Alegre, Agronomia 9090 Brazil

**Keywords:** Halicephalosis, Rhabditida, Equine pathology, Zoonosis, Public health

## Abstract

Halicephalosis is a rare cosmopolitan disease affecting equids, cattle, and humans, manifesting as meningoencephalitis. This study reports the detection of *Halicephalobus gingivalis* infection in two adult horses with neurological clinical signs in Rio Grande do Sul state, in the Southern Brazil. It underscores the pathological main findings and phylogenetic analyses. In both cases, the horses exhibited severe neurological manifestations and were euthanized due to poor prognosis. Pathological findings revealed gross brain lesions, in addition, alterations in the kidneys and lymph nodes. Histopathological examinations revealed granulomatous encephalitis, nephritis, and lymphadenitis, lesions that have been reported in human and animal infections. Molecular analyses by 28 S rRNA gene confirmed *H. gingivalis* infection and identified a genetic correlation of the isolates with Lineage 3 of the nematode, which is associated with infections in horses worldwide. This report represents the first phylogenetic description of *H. gingivalis* in Brazil and emphasizes the zoonotic significance of this parasite, particularly in occupational settings.

**Supplementary Information:**

The online version contains supplementary material available at https://doi.org/10.1007/s11259-026-11522-8.

## Background

*Halicephalobus* (Rhabditida, Panagrolaimidae) are free-living nematodes found in a wide range of environments such as decomposing organic matter (Nadler et al. 2003). *Halicephalobus gingivalis* is the only species known to cause fatal meningoencephalitis in horses and humans (Nadler et al. 2003; Lim et al. 2015). The infection occurs when larvae penetrate through skin or mucosal lesions and hematogenous and lymphatic dissemination is presumed, however, the exact pathogenesis and life cycle are not fully understood (Henneke et al. 2014). The main affected tissues include the brain and kidneys, as well as oral and nasal cavities (Eydal et al. 2012; Pintore et al. 2017). Infected individuals usually exhibit nonspecific manifestations, which lead to underdiagnosis and misinterpretation (Onyiche et al. 2018).

Halicephalosis is rarely reported in horses from Brazil, with cases in Midwestern (Sousa et al. 2018), Southeastern (Vasconcelos et al. 2007) and Northeastern (Oliveira et al. 2022). Here, we present clinical manifestations and pathological findings of systemic infection by *H. gingivalis* in two horses from Southern Brazil, with the first phylogenetic analyses of this nematode in the country.

## Case report

In July 2021 and in May 2023, an 11-year-old female Criollo horse from a stud farm in Capivari do Sul/RS/Brazil and a 12-year-old male Criollo horse from another farm in Garibaldi/RS/Brazil, respectively, were admitted to the veterinary hospital (HCV-UFRGS). Cases localities are presented in Online Resource 1. In both cases the horses presented a history of apathy, drowsiness, ataxia, reduced proprioception, grinding teeth, intestinal hypomotility and head pressing in the previous three-five days according to the owners. Due to the poor prognosis, both animals were euthanized and submitted to necropsy after 24–48 h of admission.

Fragments of organs were collected and fixed in 10% formalin solution. Afterwards, samples were processed for histology analyses with hematoxylin and eosin staining. Brain and spinal cord samples were collected for rabies virus tests through indirect immunofluorescence due to the disease obligate surveillance in reports of neurological manifestation in animals. Additionally, fragments from brain, lymph nodes and kidney were stored at −20º C for molecular diagnosis.

To prepare tissues for DNA extraction, 100 mg of brain, lymph nodes and kidney fragments were individually processed into 5 ml tubes, containing 4 zirconia microspheres and 600 µL of phosphate-alkaline buffer (PBS), by a cell and tissue disruptor L-BEADER 6^®^ (Loccus™, Cotia, SP, BR), by means of a modified protocol of 10 cycles at 3720 rpm for 40 s and 30 s of interval. Following this, genomic DNA extraction was carried out on 25 mg of the samples using the PureLink^®^ Genomic DNA Mini Kit (Invitrogen™, Carlsbad, CA, USA), following the manufacturer’s instructions for both procedures.

Samples were submitted to a polymerase chain reaction (PCR) assay targeting a portion of approximately 673 bp of 28 S rRNA gene for nematodes, using primers forward 504 (5′ CAAGTACCGTGAGGGAAAGTTG 3′) and reverse 503 (5′ CCTTGGTCCGTGTTTCAAGACG 3′) (Nadler et al. 2003). The PCR mix was composed of 26 µl of Platinum Taq DNA Polymerase (Invitrogen, Carlsbad, CA, USA), 1.5 mM of magnesium chloride (Invitrogen, Carlsbad, CA, USA), 0.2 µM of each dNTP (Invitrogen, Carlsbad, CA, USA), 10X PCR buffer (100 mM Tris–HCl, pH 9.0, 500 mM KCl), 1 µl of each forward and reverse primers, 2 µl of template DNA, and sterile ultra-pure water to complete a 25 µl reaction volume. Ultrapure water was used as negative control. Amplicons were visualized by LED transilluminator after electrophoresis in 1.5% agarose gel along with 100 bp DNA ladder (Thermo Scientific™, Carlsbad, CA, USA.

Amplicons of the expected size were purified by PureLink™ PCR Purification Kit (Invitrogen™, Carlsbad, CA, USA) and sequenced in both directions using automatic Sanger in a private company (ACTGene Soluções moleculares, Porto Alegre, BR). Consensus sequences were edited using BioEdit v. 7.2 (Hall 1999) and compared with reference sequences available on GenBank by BLAST. Two sequences from this study were aligned with homolog sequences retrieved from the GenBank online repository. The final dataset comprised 27 nucleotide sequences with 438 positions. The optimal nucleotide substitution model (Jukes and Cantor 1969) was identified using jModelTest v2.1.10 (Darriba et al. 2012). Phylogenetic analyses were conducted in MEGA11 (Tamura et al. 2021). The best phylogenetic tree was obtained after 1,000 bootstraps. Also, a matrix of pairwise distances was estimated (Tamura et al. 2004).

In case #1 brain, discrete and well-defined red 1–2 mm punctate areas were observed in the left cerebral hemisphere within the basal nuclei at the corpus striatum level. No brain gross lesions were observed in case # 2. Rabies virus detection yielded negative results. Grossly, the kidneys of Case #2 exhibited multifocal, firm, white-to-yellow nodules measuring 2–7 cm in diameter and involving both the cortex and medulla. Additionally, the right and left medial iliac lymph nodes were diffusely enlarged (10 × 6.0 × 3.5 cm), firm, and white.

Both cases exhibited moderate, multifocal perivascular cuffing, predominantly affecting arteries, along with marked chronic granulomatous arteritis characterized by a moderate, multifocal inflammatory infiltrate composed mainly of macrophages, lymphocytes, and plasma cells, with fewer multinucleated giant cells, eosinophils, fibroblasts, and fibrin. These lesions were centered on arteries and were associated with multiple cross-sections of parasitic structures in the corpus striatum, diencephalon, midbrain (case 1#), temporal cortex, hippocampus, thalamus, and cerebellum (case 2#). The arterial walls were moderately hypertrophic. Frequently there were multiple sections of elongated parasitic structures around vessels and free in the neuropil, approximately 150–200 × 15–20 μm, exhibiting a thin cuticle, platymyarian musculature, pseudocoelom, and a rhabditiform esophagus, consistent with *H. gingivalis*. Axonal spheroids were moderately present, along with mild mineralization of the vascular endothelium and mild multifocal gliosis adjacent. Additionally, discrete areas of multifocal malacia with the presence of Gitter cells were noted surrounding the affected areas. In cerebellum, there was multifocal moderate perivascular edema and occasional random gliosis with axonal spheroids. No microscopic lesions were observed in the spinal cord of either case.

**Fig. 1:**
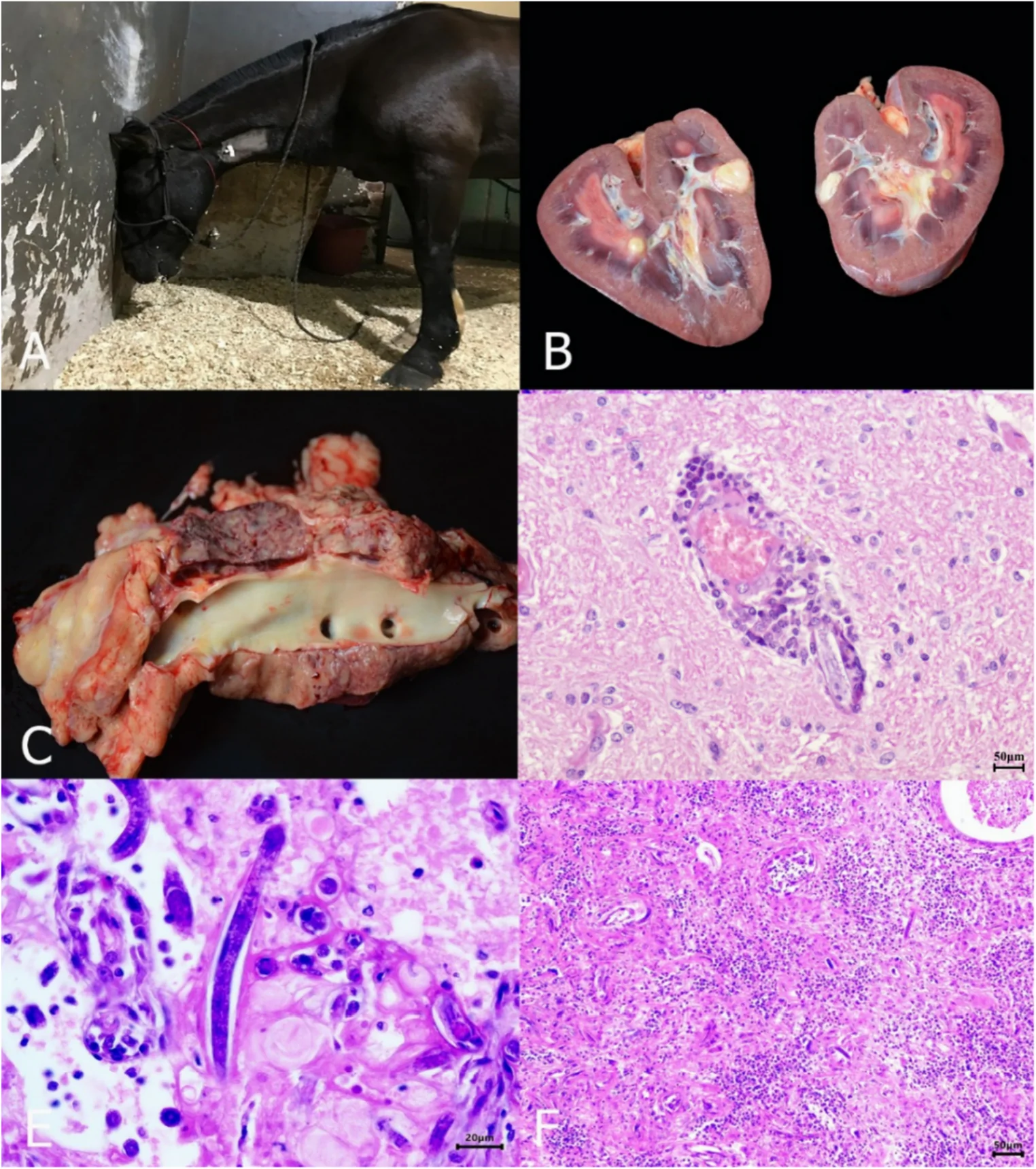
*Halicephalobus gingivalis* in horses. Clinical Manifestation: (**A**) Case #1 - horse presenting head pressing. Grossly alterations: (**B**) Case #2 - kidney. At cut surface, multifocal white to yellow, firm nodules (2–7 cm in diameter) affecting the cortex and medulla. (**C**) Case #2 - Aortic artery and right and left medial iliac lymph nodes. The lymph nodes were diffusely enlarged (10 × 6,0 × 3,5 cm), firm, and white. Histopathological findings (H&E staining): (**D**) Case #1 – brain, 40x. There was a moderate multifocal inflammatory infiltrate of macrophages, lymphocytes, plasma cells, and fewer multinucleated giant cells, eosinophils, fibroblasts, and fibrin surrounding an artery (perivascular cuffing) associated with sections of parasitic structures. The artery wall is diffusely and moderately hypertrophic. (**E**) Case #2, 60x - brain. Elongated parasite exhibiting a thin and smooth cuticle, platymyarian musculature, pseudocoelom, and a rhabditiform esophagus, consistent with *Halicephalobus* sp. (**F**) Case #2 – kidney, 40x. Parenchyma is replaced by marked inflammatory infiltrate predominantly of macrophages, eosinophils, lymphocytes, and fewer giant multinucleated cells with fibrous conjunctive tissue. Associated with this infiltrate, there were several rhabditiform larvae

Kidney nodules corresponded to focally extensive areas in which the renal parenchyma, as well as the nodal parenchyma of the affected lymph nodes, was replaced by a marked inflammatory infiltrate composed predominantly of macrophages, eosinophils, and lymphocytes, with fewer multinucleated giant cells embedded within abundant fibrous connective tissue and associated with numerous rhabditiform larvae. Histologically, lymph nodes were surrounded by an abundant inflammatory infiltrate composed of macrophages, eosinophils, plasma cells, lymphocytes, and multinucleated giant cells, associated with numerous rhabditiform larvae. Gross and histological images are presented in Fig. 1.

**Fig. 2:**
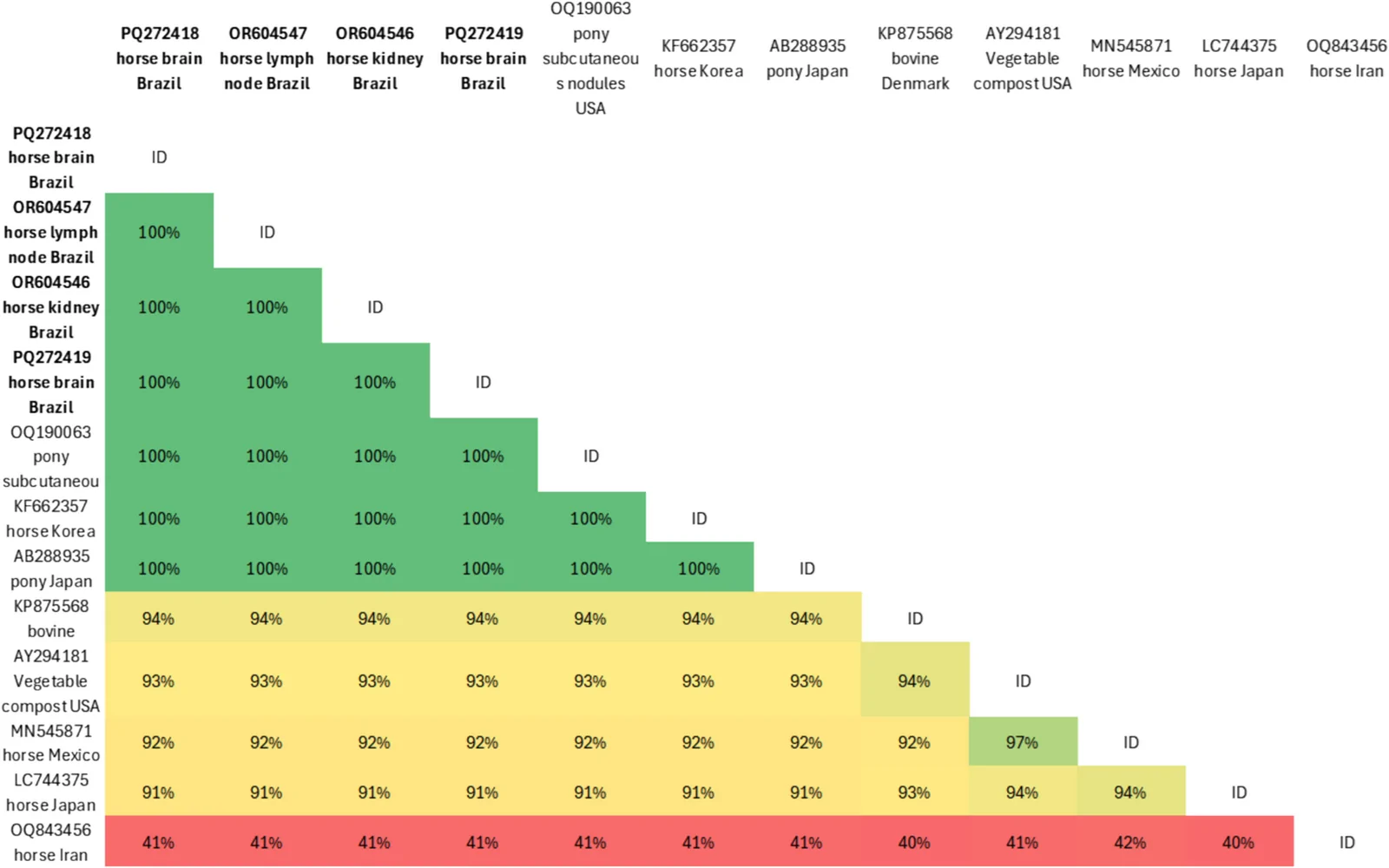
Similarity matrix of the 28 S rRNA sequences from *Halicephalobus gingivalis* from this study (bold) and sequences from GenBank. Case #1: PQ272419 (brain). Case #2: PQ272418 (brain), OR604547 (lymph node), OR604546 (kidney)

BLAST analysis revealed 100% coverage and identity with several *H. gingivalis* sequences, which were sampled from horses in the USA (OQ190063), Korea (KF662357), Japan (AB288935), and Italy (KY364905), or from environmental invertebrates (KX372288, MG051262) (Fig. 2).

**Fig. 3:**
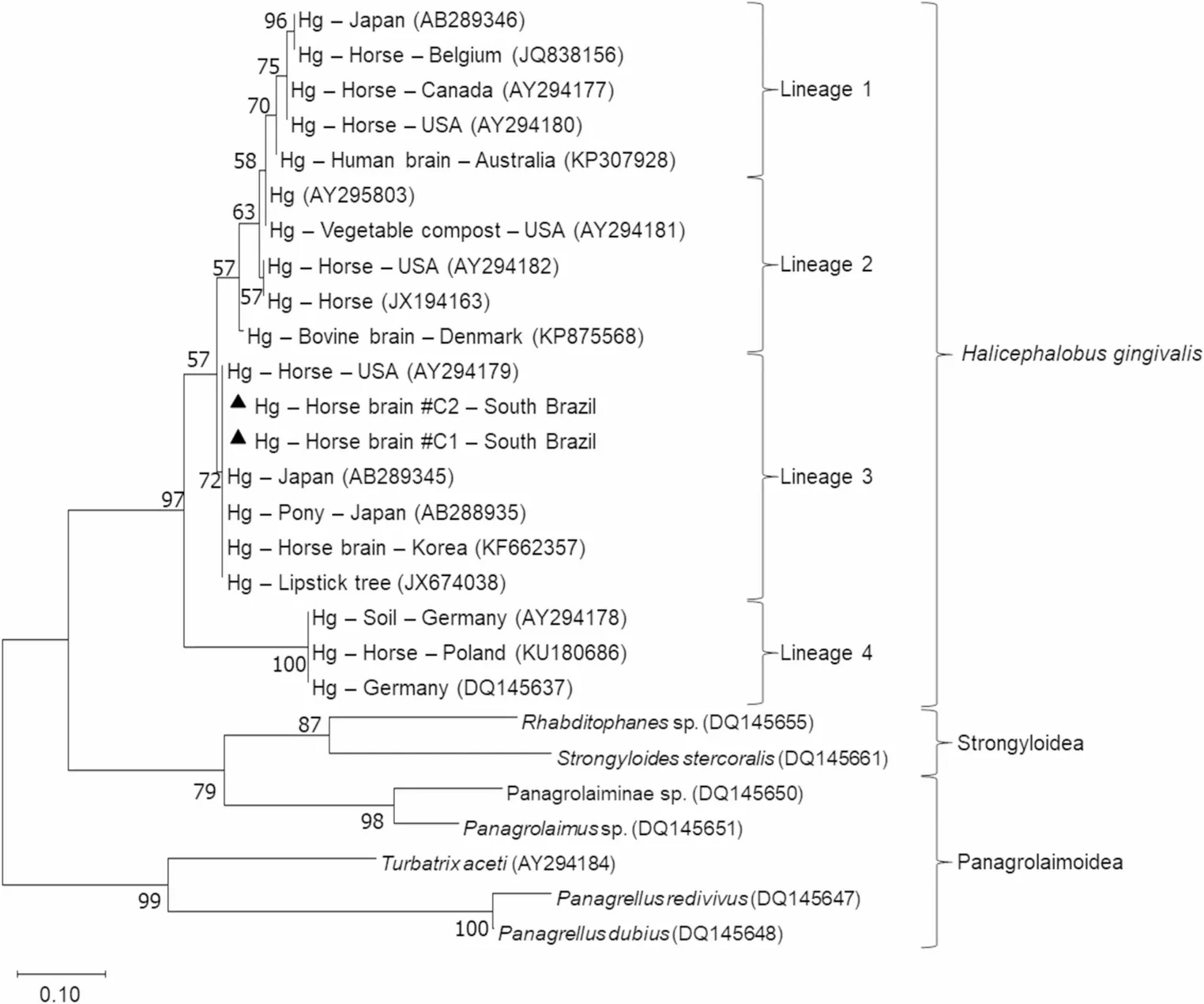
Phylogenetic tree was constructed using the Maximum-Likelihood method with 1000 bootstraps and 438 positions. Two sequences from this study (▲) and 25 homolog sequences were used. The most suitable evolutionary model was JC. Sequences from *Panagrellus* spp. were used as outgroup

The phylogenetic analysis included two sequences from the present study and 25 homologous sequences deposited in GenBank, comprising a total of 438 positions in the final dataset (Fig. 3). Both isolates from this study branched within Lineage 3 with 100% support, as previously identified by Nadler et al. (2003). Obtained sequences were deposited in GenBank under the accession numbers Case #1 - PQ272419 (brain) and Case #2 - PQ272418 (brain), OR604547 (lymph node), OR604546 (kidney).

## Discussion and conclusion

This study describes two confirmed cases of *H. gingivalis* infection in Criollo horses and provides valuable pathological and phylogenetic data from Brazil. The main findings include necrotizing encephalitis, nephritis, and lymphadenitis. Ante-mortem diagnosis of *H. gingivalis* infection is challenging due to nonspecific signs common to several infectious, parasitic and toxemic diseases important for equine health (Marcolongo-Pereira et al. 2014). Furthermore, few studies report success in treating halicephalosis with anthelmintics such as moxidectin, ivermectin and diethylcarbamazine (Pearce et al. 2001; Muller et al. 2008), which can be challenging in neurological cases due to the blood–brain barrier.

*Halicephalobus* is a free-living saprophytic nematode and has been previously reported in adult horses of 5–24 years worldwide with no gender predilection noted (Muller et al. 2008). Both horses were adults (11–12 years) from rural areas in a pasture-based system. It took an average of five days for the initial clinical signs to progress into a severe manifestation. Signs like head pressing, ataxia, and nystagmus were observed and are common in neurological infection by this nematode (Sousa et al. 2018). Also, due to poor outcomes, most of the reported cases led to death or euthanasia. Systemic clinical signs (e.g. lameness, dermatitis and posthitis) depend on the localization of the parasite and may have a blander presentation (Oliveira et al. 2022; Sousa et al. 2024).

The infection by *H. gingivalis* is suspected to occur through oral-nasal mucosa or skin wounds where larvae reach blood or lymph vessels and spread to several organs based on occurrence of oral and nasal wounds and common infection sites (Onyiche et al. 2018). The brain is the most involved site of infection, though parasitic infection in kidney and lymph nodes is frequently reported (Pearce et al. 2001). Macroscopic lesions are not frequently described in this disease (Henneke et al. 2014; Sousa et al. 2018), in contrast to the present study where discreet lesions were observed in multiple sections of the brain in case #1, and severe lesions in lymph nodes and kidneys in case #2. Regarding histological lesions, the presence of parasitic structures in the perivascular areas suggests a hematogenous spread of the parasite (Bryant et al. 2006). The predominant infiltrate cells observed were macrophages, lymphocytes, eosinophils, giant cells associated with rhabditiform larvae. *H. gingivalis* nematodes are usually associated with perivascular inflammatory infiltrate (Sousa et al. 2018), causing eosinophilic encephalitis in horses (Zoll et al. 2018). The characteristic morphology of the rhabditiform esophagus, along with molecular analysis, facilitated the identification of *H*. *gingivalis*, eliminating the diagnosis of other nematodes that can induce encephalitis in horses, such as *Strongylus vulgaris* and *Setaria digitata* (Bryant et al. 2006; Furr 2015).

*Halicephalobus* is a zoonotic nematode associated with a fatal human meningoencephalomyelitis (Ondrejka et al. 2010). The neurological and histological features are similar to those described in horses and observed in this study cases (Lim et al. 2015). Human cases remain extremely rare, and none have been reported in Brazil (Ondrejka et al. 2010; Lim et al. 2015; Monoranu et al. 2015). Nevertheless, neurotropic parasitic infections diagnosis is also challenging in human medicine (Shahrivar et al. 2025), therefore it is important to consider halicephalosis as possibility in areas where animal infection have been reported.

Phylogenetic analyses are very scattered, and no previously molecular studies were carried out in Brazil. The *H. gingivalis isolates* presented here belonged to Lineage 3 according to phylogenetic analyses of the 28 S rRNA gene. This lineage includes isolates from distinct world regions (Japan, Korea, Italy and USA) revealing no geographical correlation between the nematode genetics (Pintore et al. 2017). Further and more robust phylogenetic analyses could provide important information about correlations between lineages and pathogenic and zoonotic potentials (Nadler et al. 2003; Onyiche et al. 2018), since isolates from human infection revealed to belong to Lineage 1 (Monoranu et al. 2015). The limited quantity of sequences and genes deposited in GenBank restrict a better epidemiologic and phylogenetic overview of *H. gingivalis* worldwide.

This report provides the first molecular analyses related to a new geographic site and pathological features of infection in horses by *H. gingivalis* in Brazil. The findings emphasize the need for differential diagnosis and surveillance of neurological disorders in horses. Further studies are needed to understand epidemiology as well enhance phylogenetic data generation to support ecological and genomic associations.

## Supplementary Information

Below is the link to the electronic supplementary material.

Supplementary Material 1 (DOCX 197 KB)

## Data Availability

No datasets were generated or analysed during the current study.
