# Supplementary material for: Multisystemic and cerebral infection associated with Halicephalobus gingivalis in Brazilian Criollo horses
Source: Vet Res Commun. 2026 Sep 29;50(6):602. doi: 10.1007/s11259-026-11522-8 (PMC13624022; doi:10.1007/s11259-026-11522-8)
Supplement: Supplementary file 1 [file 11259_2026_11522_MOESM1_ESM.docx]

**Multisystemic and cerebral infection associated with *Halicephalobus gingivalis* in Brazilian Criollo horses**

Laura Berger, Joanna V. Z. Echenique, Vinícius Baggio-Souza, Renata Fagundes-Moreira, Paula R. Ribeiro, Francisca M. S. Barbosa, Eryca C. Lamego, Flávia U. Bueno, Saulo P. Pavarini, Luciana Sonne, Welden Panziera, João F. Soares

***Corresponding author**: joao.soares@ufrgs.br (J.F. Soares), <https://orcid.org/0000-0002-7967-6569>

Laboratório de Protozoologia e Rickettsioses Vetoriais – ProtozooVet, Faculdade de Veterinária, Universidade Federal do Rio Grande do Sul, Avenida Bento Gonçalves, 9090, Agronomia, Porto Alegre, Brazil

**Fig.1** Geographical area in the state of Rio Grande do Sul (RS) in southern Brazil (light yellow). The reported cases were from two municipalities of RS (red dots): Capivari do Sul (case #1; 30º08'42" S 50º30'53" W) and Garibaldi (case #2; 29º15'21" S 51º32'02" W)
